# Real-Life Outcomes of First-Line Palliative Chemoimmunotherapy in Oesophago-Gastric Cancers: A Multi-Centre Retrospective Cohort Study

**DOI:** 10.3390/cancers18101522

**Published:** 2026-05-09

**Authors:** James Birch-Ford, Ben Crosby, Grace Langford, Alexandra Johnson, Helen Wong, Shobha Silva, Amy Jackson, Roopa Kurup, Joachim Chan, Alia Alchawaf, Tom Crosby

**Affiliations:** 1 Whiston Hospital, Mersey and West Lancashire Teaching Hospitals NHS Trust, Prescot L35 5DR, UK; james.birchford@merseywestlancs.nhs.uk; 2 The Clatterbridge Cancer Centre, Clatterbridge Cancer Centre NHS Foundation Trust, Liverpool L7 8YA, UK; james.birchford@merseywestlancs.nhs.uk (J.B.-F.); alexandra.johnson11@nhs.net (A.J.); helen.wong1@nhs.net (H.W.); shobha.silva@nhs.net (S.S.); amy.jackson29@nhs.net (A.J.); roopa.kurup@nhs.net (R.K.); joachim.chan@nhs.net (J.C.); 3 Velindre Cancer Centre, Velindre University NHS Trust, Cardiff CF15 7QZ, UK; ben.crosby2@wales.nhs.uk (B.C.); grace.langford@wales.nhs.uk (G.L.)

**Keywords:** chemoimmunotherapy, pembrolizumab, nivolumab, oesophago-gastric cancers, oesophageal cancer, gastro-oesophageal junction cancer, gastric cancer, immunotherapy toxicity, gastrointestinal cancers

## Abstract

**Background:** Chemoimmunotherapy has improved survival compared with chemotherapy alone in phase III trials of advanced oesophago-gastric (OG) cancers; however, real-world UK data under National Institute for Health and Care Excellence (NICE) eligibility criteria remain limited. This study evaluated the effectiveness and safety of first-line pembrolizumab- or nivolumab-based chemoimmunotherapy in routine clinical practice. **Methods:** We conducted a retrospective, multi-centre cohort study of patients with unresectable or metastatic oesophageal, gastro-oesophageal junction, or gastric cancers treated with first-line chemoimmunotherapy at two UK tertiary centres between April 2021 and July 2024. Clinical, laboratory, radiological, and toxicity data were collected. Radiological outcomes were based on retrospective review of reports issued by consultant radiologists during routine clinical care. Overall survival (OS) and progression-free survival (PFS) were analysed using the Kaplan–Meier method, with exploratory analyses of prognostic factors. **Results:** Seventy-six patients were included (59.2% ≥ 65 years; 81.6% adenocarcinoma; 71.1% metastatic). At a median follow-up of 11 months, 46 deaths had occurred. Median OS was 16.0 months (95% CI: 11.0–20.9), and median PFS was 8.0 months (95% CI: 6.8–9.2). Disease control occurred in 80.3% of patients and was associated with improved OS compared with progressive disease (17.0 vs. 4.0 months; *p* < 0.001). Survival outcomes did not differ significantly by tumour site, histology, or immunotherapy agent. Immunotherapy-related adverse events occurred in 31 patients (40.8%), with grade ≥ 3 toxicities in 13.2% and two treatment-related deaths. Exploratory analyses suggested potential associations between survival and baseline lymphocyte count and neutrophil–lymphocyte ratio, although these did not reach statistical significance. **Conclusions:** In this real-world UK multi-centre cohort, first-line chemoimmunotherapy demonstrated survival outcomes comparable to pivotal clinical trials, with manageable toxicity. These findings support the use of chemoimmunotherapy in routine practice. Prospective collaborative studies incorporating robust biomarker evaluation are warranted to optimise patient selection and better define predictors of response and toxicity.

## Simple Summary

Chemoimmunotherapy combining chemotherapy with immune checkpoint inhibitors such as pembrolizumab or nivolumab has improved survival for patients with advanced oesophago-gastric cancers in clinical trials. However, real-world outcomes in patients treated according to UK National Institute for Health and Care Excellence (NICE) criteria remain limited. This multi-centre UK retrospective study evaluated 76 patients treated with first-line palliative chemoimmunotherapy. At a median follow-up of 11 months, the median overall survival was 16 months and median progression-free survival was 8 months. Survival outcomes were similar across tumour sites, histological subtypes, and immunotherapy regimens. Immune-related adverse events occurred in 40.8% of patients, including two treatment-related deaths. Exploratory biomarker analyses suggested potential associations between survival and baseline lymphocyte count and neutrophil–lymphocyte ratio, although these did not reach statistical significance. These findings demonstrate real-world survival outcomes and toxicity profiles that are comparable to those seen in pivotal clinical trials, supporting the use of chemoimmunotherapy in routine clinical practice. Further prospective multi-centre collaborative studies are needed to validate these findings and identify predictive biomarkers of treatment response and toxicity.

## 1. Introduction

Oesophago-gastric (OG) cancers—including oesophageal, gastro-oesophageal junction (GOJ), and gastric malignancies—account for approximately 4% of all cancer diagnoses in the United Kingdom [1]. These cancers are often diagnosed at an advanced stage and are associated with a poor prognosis, with 5-year survival rates ranging from 18% to 23.9% [2,3,4]. Over the past four decades, the incidence of OG cancers—particularly oesophageal adenocarcinoma (AC)—has risen, contributing to an increasing clinical burden [5,6]. Despite potentially curative treatments such as surgery or radical radiotherapy, recurrence remains common, and many patients ultimately develop locally recurrent or metastatic disease [7,8,9].

Historically, first-line treatment for advanced HER2-negative disease consisted of fluoropyrimidine and platinum-based chemotherapy, with trastuzumab reserved for the minority of patients with HER2-positive GOJ or gastric AC [10,11]. However, most patients experience disease progression and are unable to access subsequent lines of therapy [12]. As a result, outcomes remain poor, with 5-year survival rates for metastatic disease estimated at 4–10% [3,4]. These limitations have driven the development of novel therapeutic strategies.

Recent advances in targeted therapies and immunotherapy (IO) have transformed the treatment landscape for advanced OG cancers [13,14,15,16]. Immune checkpoint inhibitors enhance anti-tumour immune responses by targeting inhibitory pathways that suppress T-cell activity. Pembrolizumab and nivolumab are anti-programmed cell death protein 1 (anti-PD-1) monoclonal antibodies that block interactions with programmed death ligand 1 (PD-L1) and 2 (PD-L2). This interaction inhibits immune checkpoint signalling and enhances T-cell-mediated anti-tumour immunity [17,18]. PD-L1 expression is observed in approximately 50–60% of OG adenocarcinomas and 20–80% of oesophageal squamous cell carcinomas (SCCs), providing a potential predictive biomarker for response to immune checkpoint inhibition [17]. Several landmark phase III randomised controlled trials have demonstrated improved outcomes with first-line chemo-immunotherapy (chemo-IO) compared with chemotherapy alone in advanced OG cancers. These include KEYNOTE-590 [13], CheckMate-648 [14], KEYNOTE-859 [15], and CheckMate-649 [16], all of which reported improvements in overall survival (OS) and progression-free survival (PFS) with the addition of pembrolizumab or nivolumab to chemotherapy (Table 1). These trials established chemo-IO as a new standard of care in appropriately selected patients, and subsequent approvals by the National Institute for Health and Care Excellence (NICE) have incorporated PD-L1 expression thresholds to guide treatment selection [19,20,21,22].

Despite survival benefits, immune checkpoint inhibitors are associated with immune-related adverse events (irAEs), which result from off-target immune activation and may affect multiple organ systems. Although many irAEs are mild, severe toxicities can occur and may be life-threatening. Reported rates of grade 1–2 toxicities (graded as per Common Terminology Criteria for Adverse Events [23]) vary widely across trials, ranging from 19.2% in KEYNOTE-859 to 93.7% in CheckMate-649 [15,16]. Grade 3–5 toxicities are less common but clinically significant, with fatal events reported in both KEYNOTE-590 and KEYNOTE-859 [13,15]. Careful monitoring and early management of irAEs are therefore critical components of clinical practice.

Although pivotal phase III trials have demonstrated improved outcomes with first-line chemo-IO, their generalisability to routine clinical practice remains uncertain. Clinical trial populations are typically highly selected according to strict eligibility criteria, whereas real-world patients are often older, have greater comorbidity, and present with more heterogeneous disease characteristics. Consequently, treatment outcomes and toxicity profiles observed in routine clinical practice may differ from those reported in randomised trials. Furthermore, real-world evidence evaluating first-line chemo-IO in upper gastrointestinal malignancies—particularly within UK populations treated according to NICE eligibility criteria—remains limited [24].

Therefore, this multi-centre retrospective study aimed to evaluate real-world outcomes of first-line palliative chemo-IO in patients with advanced OG cancers. We assessed overall survival and progression-free survival, compared outcomes with clinical trial data, evaluated the incidence of irAEs, and investigated exploratory biomarkers associated with survival.

## 2. Methods

### 2.1. Study Design and Patient Population

This retrospective, multi-centre observational cohort study evaluated outcomes in patients with advanced oesophageal, GOJ, and gastric cancers treated with first-line palliative chemo-IO. Patients were identified from prospectively maintained electronic health records at two UK tertiary cancer centres: Clatterbridge Cancer Centre and Velindre Cancer Centre. This study was conducted as a service evaluation in accordance with institutional clinical governance policies, and formal research ethics committee approval was not required in line with UK Health Research Authority guidance.

All patients who received first-line palliative chemo-IO with the immune checkpoint inhibitors pembrolizumab or nivolumab, in combination with platinum- and fluoropyrimidine-based chemotherapy, from local implementation of these regimens between April 2021 and July 2024, were eligible for inclusion. First-line palliative treatment was defined as systemic therapy administered for unresectable locally advanced or metastatic disease. Eligible patients had histologically confirmed unresectable locally advanced or metastatic oesophageal, GOJ, or gastric malignancy and were treated with chemo-IO in the first-line palliative setting.

Patients accessed chemo-IO according to National Institute for Health and Care Excellence (NICE) guidance. Pembrolizumab in combination with platinum- and fluoropyrimidine-based chemotherapy was approved for patients with oesophageal squamous cell carcinoma (SCC) and adenocarcinoma (AC) with PD-L1 combined positive score (CPS) ≥ 10% [19], and for GOJ/gastric cancer with PD-L1 CPS ≥ 1% [20]. Nivolumab in combination with platinum- and fluoropyrimidine-based chemotherapy was approved for patients with oesophago-gastric adenocarcinoma (OG AC) with PD-L1 CPS ≥ 5% [21] and for oesophageal SCC with PD-L1 tumour proportion score (TPS) ≥ 1% [22].

Chemotherapy regimens included oxaliplatin with capecitabine (CAPOX), oxaliplatin with infusional 5-fluorouracil (FOLFOX), carboplatin with capecitabine, and cisplatin with capecitabine. Regimens were administered according to institutional practice and clinician discretion. The proportion of patients receiving each regimen is reported in the baseline characteristics (Table 2). Treatment was delivered as part of routine clinical practice, with no predefined restrictions based on performance status.

Patients who had previously received radical treatment (e.g., surgery or chemoradiotherapy) and subsequently developed recurrent or metastatic disease were included if chemo-IO represented their first-line treatment in the palliative setting. Similarly, patients who had received neoadjuvant chemotherapy but later developed disease progression that precluded curative resection were included if they proceeded to first-line palliative chemo-IO. Patients were excluded if they received immunotherapy outside the first-line palliative setting or if insufficient clinical data were available for analysis.

### 2.2. Data Collection

Clinical data were collected retrospectively from electronic medical records and included demographic characteristics, tumour histology, primary tumour site, stage at diagnosis, treatment regimen, Eastern Cooperative Oncology Group (ECOG) performance status, and baseline laboratory parameters. Baseline laboratory values included haemoglobin, white cell count, neutrophil count, lymphocyte count, monocyte count, platelet count, and albumin levels. Baseline laboratory parameters were defined as the most recent values obtained prior to treatment initiation (range 2–14 days). Derived inflammatory markers, including neutrophil-to-lymphocyte ratio, were calculated where applicable.

PD-L1 expression was assessed using the combined positive score (CPS) with the 22C3 pharmDx assay on the DAKO Autostainer AS L55 platform at both centres. While different companion diagnostic assays have been used in clinical trials of immune checkpoint inhibitors, NICE guidance does not mandate assay-specific testing [19,20,21,22]. Use of 22C3 CPS-based assessment reflects routine UK practice and supports the applicability of these results to real-world NHS settings.

Data on treatment exposure, immune-related adverse events (irAEs), radiological disease progression, last follow-up, cause of death where available, and survival status were also collected. Immune-related adverse events were graded according to the National Cancer Institute Common Terminology Criteria for Adverse Events version 5.0 (CTCAE v5.0). Follow-up duration was calculated from treatment initiation to last recorded clinical contact.

### 2.3. Outcomes and Definitions

The primary outcomes of the study were overall survival (OS) and progression-free survival (PFS). Secondary outcomes included objective response rate, disease control rate, and incidence of immune-related adverse events.

Overall survival was defined as the time from initiation of chemo-IO to death from any cause. Patients who were alive at last follow-up were censored at that date. Progression-free survival was defined as the time from initiation of chemo-IO to radiologically confirmed disease progression or death from any cause, consistent with standard clinical trial definitions. Deaths occurring prior to documented disease progression were considered PFS events. This included deaths related to treatment toxicity, intercurrent illness, or other causes. Patients without documented progression or death were censored at last follow-up.

Radiological response and disease progression were assessed using cross-sectional imaging performed as part of routine clinical care, typically at 8- to 12-week intervals. Radiological outcomes were determined through retrospective review of radiology reports issued by consultant radiologists during standard clinical care. Formal RECIST criteria were not applied, reflecting real-world clinical practice. Disease control was defined as complete response, partial response, or stable disease as documented in radiology reports.

### 2.4. Statistical Analysis

Statistical analysis was performed by a qualified statistician using IBM SPSS Statistics for Windows, Version 29.0 (IBM Corp., Armonk, NY, USA). Continuous non-parametric data were compared using the Mann–Whitney U test or Kruskal–Wallis test, as appropriate. Categorical variables were compared using the chi-square test or Fisher’s exact test.

Survival outcomes were analysed using the Kaplan–Meier method, with comparisons between groups performed using the log-rank test. Cox proportional hazard regression analysis was performed to evaluate prognostic factors associated with survival outcomes. Variables included in multivariable analysis were selected based on clinical relevance and univariable significance (*p* < 0.10).

Missing data were handled using complete case analysis, and no imputation was performed. Two-sided *p*-values < 0.05 were considered statistically significant.

Given the modest sample size, analyses evaluating potential prognostic biomarkers were considered exploratory and hypothesis-generating.

### 2.5. Subgroup Analysis

Pre-specified subgroup analyses were conducted according to PD-L1 expression, histology, and primary tumour site. Additional exploratory analyses were performed to evaluate the association between baseline clinical and laboratory parameters and survival outcomes.

## 3. Results

### 3.1. Patient Demographics

In total, we identified 76 patients who were eligible for inclusion and subsequent data collection (Table 2); 41 (55.2%) were treated at the Clatterbridge Cancer Centre and 34 (44.8%) were treated at the Velindre Cancer Centre; 57 (75%) patients were male and 19 (25%) patients were female; and 45 (59.2%) patients were older than the age of 65. Chemo-IO was used as first-line palliative treatment in 39 (51.3%) patients with oesophageal cancer, 31 (40.8%) patients with GOJ cancer and 6 (7.9%) patients with gastric malignancies. Histologically, 62 (81.6%) tumours were AC and 14 (18.4%) tumours were SCC. Pembrolizumab was the IO agent for 61 (80.3%) patients and 15 (19.7%) received nivolumab. Regarding performance status (PS), 24 (36.6%) patients were PS0, 47 (61.8%) patients were PS1 and 5 (6.6%) were PS2.

**Table 2:** Demographic data from the 76 identified oesophago-gastric cancer patients treated with palliative chemoimmunotherapy (chemo-IO) at Clatterbridge and Velindre Cancer Centres in the UK.

Patient Demographic	*N*	%
**Sex**		
Female	19	25.0%
Male	57	75.0%
**Age group**		
<65	31	40.8%
≥65	45	59.2%
**Tumour group**		
Oesophageal	39	51.3%
GOJ	31	40.8%
Gastric	6	7.9%
**Histology**		
Adenocarcinoma	62	81.6%
Squamous cell carcinoma	14	18.4%
**ECOG performance status**		
0	24	31.6%
1	47	61.8%
2	5	6.6%
**Tumour stage**		
T1	2	2.6%
T2	5	6.6%
T3	31	40.8%
T4	34	44.7%
TX	4	5.3%
**Nodal stage**		
N0	10	13.2%
N1	18	23.7%
N2	24	31.6%
N3	20	26.3%
NX	4	5.3%
**Metastatic stage**		
M0	22	28.9%
M1	54	71.1%
**Overall stage**		
I	1	1.3%
II	7	9.2%
III	14	18.4%
IV	54	71.1%
**Immunotherapy agent**		
Nivolumab	15	19.7%
Pembrolizumab	61	80.3%
**Chemotherapy regimens**		
Oxaliplatin and capecitabine (CAPOX)	65	85.5%
Carboplatin and capecitabine	8	10.5%
Oxaliplatin and 5-FU infusion (FOLFOX)	2	2.6%
Cisplatin with capecitabine	1	1.3%

As expected in the palliative setting, patients with stage IV, III, II and I disease numbered 54 (71.1%), 14 (18.4%), 7 (9.2%) and 1 (1.3%), respectively. Additionally, 54 (71.1%) patients had visceral metastatic spread. Regarding radiological nodal staging, 62 (81.6%) patients had lymphatic involvement, 10 (13.2%) patients had no lymphatic involvement and 4 (5.3%) had NX nodal grading. In terms of tumour staging, 34 (44.7%) patients were T4, 31 (40.8%) were T3, 5 (6.6%) were T2, 2 (2.6%) were T1 and 4 (5.3%) were TX.

On average, patients received six cycles of chemotherapy and eight cycles of IO. There were various reasons why chemo-IO was stopped, including disease progression, toxicity, physiological deterioration and patient choice. All patients who experienced irAEs were managed with input from specialist toxicity services.

All patients had histologically assessed PD-L1 values with an average CPS of 29.0 (range: 5–100). The average PD-L1 CPS of oesophageal (*n* = 36) and GOJ/gastric (*n* = 25) patients treated with pembrolizumab was 36.6 and 27.6, respectively. In OG adenocarcinoma patients (*n* = 15) treated with nivolumab, the average PD-L1 CPS was 15.3. No oesophageal SCC patients were treated with nivolumab. Of the 62 patients with AC, 2 were mismatch repair (MMR)-deficient, 50 were MMR-proficient and 10 patients had unknown MMR status.

### 3.2. Patient Outcomes and Toxicity

**Figure 1:**
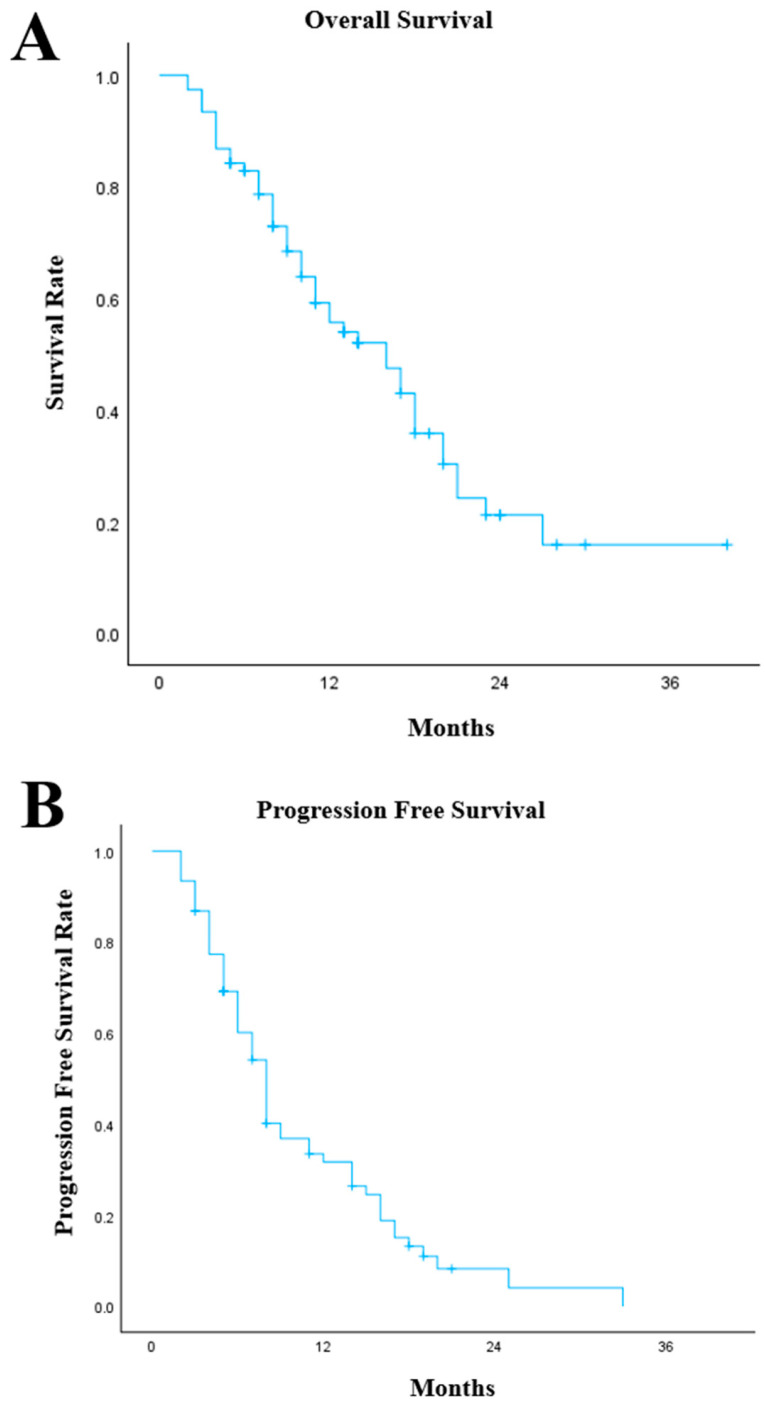
Kaplan–Meier graphs illustrating (**A**) the overall survival of the 76 patients with oesophago-gastric cancers treated with chemoimmunotherapy with pembrolizumab or nivolumab from the start date of treatment, (**B**) the progression-free survival of the 76 patients with oesophago-gastric cancers treated with chemoimmunotherapy with pembrolizumab or nivolumab from the start date of treatment.

In our cohort, the median OS was 16 months (95% Confidence Interval [CI]: 11.0–20.9) from the start of treatment with chemo-IO (Figure 1A). Patients with oesophageal (*n* = 39), GOJ (*n* = 31) and gastric (*n* = 6) cancers had similar outcomes with median OS of 14 months (95% CI: 8.5–19.5), 16 months (95% CI: 8.2–23.8) and 16 months (95% CI: 0–36.0), respectively (*p* = 0.719). Histologically, patients with SCC (*n* = 14) had a median OS of 18 months (95% CI: 10.0–26.0) compared with AC (*n* = 62) with a median OS of 13 months (95% CI: 7.2–18.8, *p* = 0.180). Within the pembrolizumab cohort (*n* = 61), the median OS was 16 months (95% CI: 10.8–21.2) similar to the nivolumab cohort (*n* = 15) with a median OS of 16 months (95% CI: 5.3–26.7). At the time of data cut-off, 46 deaths had occurred. The median follow-up duration was 11 months from initiation of chemoimmunotherapy.

The median PFS was 8 months (95% CI: 6.8–9.2) from the start of chemo-IO treatment (Figure 1B). There was no significant difference between oesophageal, GOJ and gastric cancers with median PFS of 8 months (95% CI: 7.0–9.0), 6 months (95% CI: 4.0–8.0) and 8 months (95% CI: 3.1–12.9), respectively (*p* = 0.780). Moreover, the median PFS was similar between tumour histologies (*p* = 0.336), AC (*n* = 62) had a median PFS of 8 months (95% CI: 6.6–9.4) and SCC (*n* = 14) had a median PFS of 8 months (95% CI: 6.4–9.6). Patients treated with pembrolizumab (*n* = 61) had a median PFS of 8 months (95% CI: 7.0–9.0) compared with 7 months (95% CI: 5.0–9.0) within the nivolumab population.

**Figure 2:**
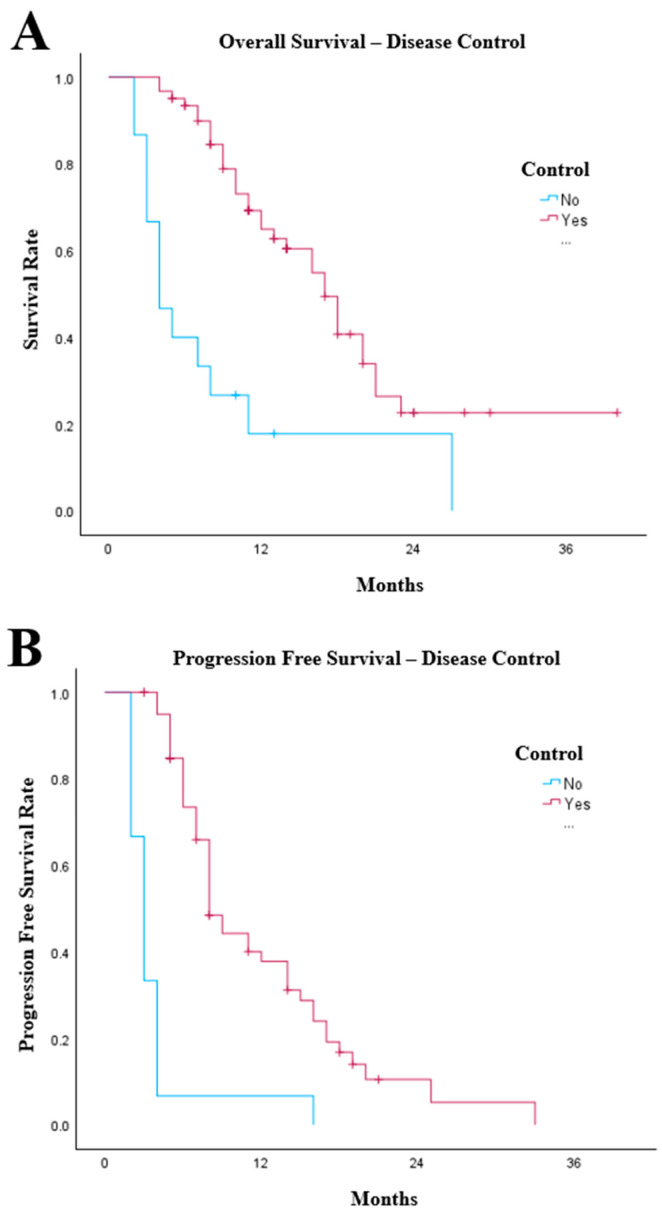
Kaplan–Meier graphs illustrating (**A**) the overall survival of patients with oesophago-gastric cancer treated with chemoimmunotherapy separated by whether they achieved disease control (*n* = 61) or disease progression (*n* = 15, *p* < 0.001), (**B**) the progression free survival of patients with oesophago-gastric cancer treated with chemoimmunotherapy separated by whether they achieved disease control (*n* = 61) or disease progression (*n* = 15, *p* < 0.001).

Disease control (complete response, partial response or stable disease) was observed in 61 patients (80.3%). Patients who had disease control with chemo-IO had a median OS of 17 months (95% CI: 14.0–20.0) compared with 4 months (95% CI: 2.1–5.9) in patients who progressed on chemo-IO (*p* < 0.001, Figure 2A). Patients who achieved radiological response to chemo-IO (*n* = 61) had significantly longer PFS than those who did not respond (*n* = 15; *p* < 0.001). Median PFS was 8 months (95% CI: 6.8–9.2) compared with 3 months (95% CI: 2.3–3.7) in patients with immediate radiological progression (Figure 2B).

In univariate Cox regression analysis for OS, greater numbers of IO cycles were associated with improved overall survival (HR 0.84, 95% CI 0.78–0.91, *p* < 0.001). Additionally, male patients had poorer outcomes than female patients following chemo-IO. (HR: 2.69, 95% CI: 1.19–6.06, *p* = 0.017).

**Figure 3:**
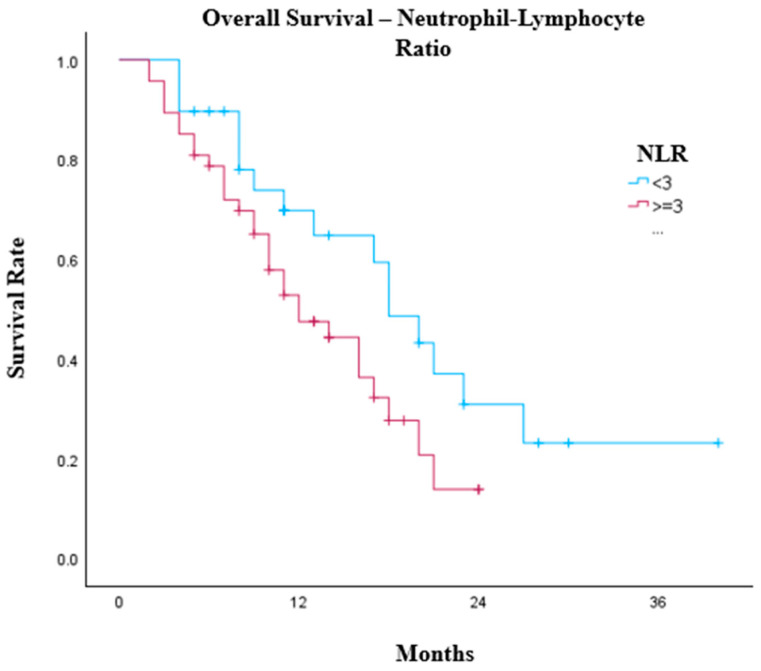
Kaplan–Meier graph illustrating the OS of oesophago-gastric patients treated with first-line chemoimmunotherapy separated by pre-treatment neutrophil–lymphocyte ratio < 3 (*n* = 29) and ≥3 (*n* = 47, *p* = 0.057).

The occurrence of colitis had a weak association with worse survival outcomes (*p* = 0.087); however, there was no survival benefit with age or any other irAEs. PD-L1 CPS had no impact on OS (*p* = 0.308). In exploratory analyses, pre-treatment lymphocyte count (*p* = 0.064) and neutrophil–lymphocyte ratio (NLR, *p* = 0.075) demonstrated non-significant strong trends towards association with overall survival, with lymphocytes > 1.0 × 10^9^/L (*p* = 0.099) and lower NLR values—particularly at thresholds of <4 (*p* = 0.169) and <3 (*p* = 0.065)—generally associated with improved survival outcomes (Figure 3). The median OS of patients with a pre-treatment NLR < 3 was 18 months (95% CI: 14.1–21.9) compared with 12 months (95% CI: 7.5–16.4, *p* = 0.057). There were no other significant or borderline pre-treatment blood test results, including white cell count, monocytes, haemoglobin, platelets or albumin, that suggested improved survival benefit within our cohort.

Again, in univariate Cox regression analysis to assess for features associated with PFS, more IO cycles were associated with prolonged duration of treatment prior to disease progression (HR: 0.90, 95% CI: 0.851–0.952, *p* < 0.001). Gender, age and the irAEs had no significant impact in the PFS of patients.

irAEs occurred in 31 patients (40.8%), with a total of 34 toxicity events recorded, with 3 patients who experienced multiple toxicities (Table 3). There were six (7.9%) cases of hepatitis and colitis, five (6.6%) cases of hypothyroidism, four (5.3%) cases of neuropathy and dermatitis, two (2.6%) cases of nephritis and pneumonitis and one (1.3%) case of each out of myositis, meningoencephalitis, myalgia and endocrinopathy. There was 1 (1.3%) case of grade 4 toxicity, 7 (9.2%) cases of grade 3 toxicity, 14 (18.4%) cases of grade 2 toxicity and 10 (13.2%) cases of grade 1 toxicity. There were two (2.6%) fatal (grade 5) immune-related adverse events: hepatitis and meningoencephalitis.

**Figure 4:**
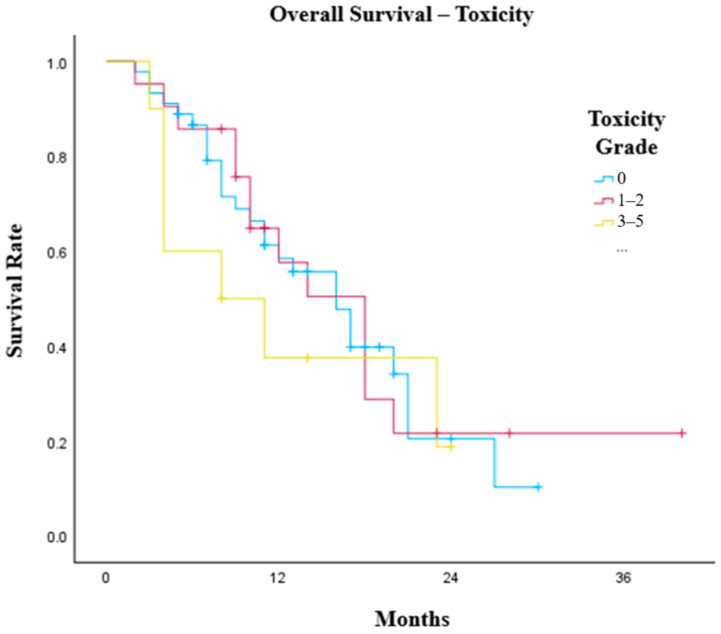
Kaplan–Meier graph illustrating the OS of oesophago-gastric patients treated with first-line chemoimmunotherapy separated by the presence and grade of immunotherapy-related toxicity. No significance between grade 0 and grade 1–2 (*p* = 0.834) or grade 0 and grade 3–5 (*p* = 0.439).

No significant difference was observed in OS by the presence or grade of irAEs. Patients who did not experience IO toxicity had an OS of 16 months (95% CI: 11.8–20.2) compared with 14 months (95% CI: 8.6–19.4) in patients who developed irAEs (*p* = 0.853). Moreover, patients with grade 1 toxicity had a median OS of 10 months (95% CI: 8.7–11.3), those with grade 2 toxicity had a median OS of 18 months (95% CI: 14.8–21.2), those with grade 3 toxicity had a median overall survival of 11 months (95% CI: 0–29.0) and those with grade 5 toxicity had a median OS of 3 months (*p* = 0.156). In grouped analysis (Figure 4), there was no survival benefit between patients who did not experience irAEs compared with patients who developed grade 1 or 2 toxicity (*p* = 0.834) and patients who developed grade 3–5 toxicity (*p* = 0.439).

Additionally, no difference was observed in PFS by the presence or grade of irAEs. Patients who experienced IO toxicity had a median PFS of 8 months (95% CI: 7.0–9.0) compared with 7 months (95% CI: 5.4–8.6) for those who did not experience toxicity (*p* = 0.733). There was no significant difference in PFS between grades of toxicity (*p* = 0.796); the median PFS of patients with grade 1, 2, 3 and 5 toxicities was 8 months (95% CI: 6.8–9.2), 8 months (95% CI: 5.7–10.3), 11 months (95% CI: 0–29.0) and 3 months, respectively.

## 4. Discussion

In this multi-centre cohort of 76 patients with oesophageal, GOJ, and gastric cancers treated with first-line palliative chemo-IO, the median OS was 16 months.

This survival outcome is comparable to results from pivotal phase III trials that established chemo-IO as standard first-line therapy for advanced upper gastrointestinal malignancies. In the KEYNOTE-590 [13] study, pembrolizumab combined with chemotherapy improved median OS to 12.3 months compared with 9.8 months with chemotherapy alone (HR 0.72). Similarly, CheckMate-649 [16] demonstrated a median OS of 12.8 months with nivolumab plus chemotherapy versus 11.6 months with chemotherapy alone in the overall randomised population (HR 0.89). More recently, KEYNOTE-859 [15] reported a median OS of 12.9 months with pembrolizumab plus chemotherapy compared with 11.5 months with chemotherapy alone (HR 0.78) in patients with HER2-negative advanced gastric or GOJ AC. Collectively, these trials reported median OS values of approximately 12–14 months. The 16-month survival observed in our cohort is therefore broadly consistent with outcomes reported in clinical trials, despite the greater clinical heterogeneity typical of real-world populations. However, in the context of IO, median OS may not fully represent the survival benefit of patients; a proportion of these patients achieve durable long-term responses leading to a characteristic ‘long tail’ in survival curves and improved landmark survival at later timepoints. In smaller, real-world cohorts, this effect may be less apparent and not fully reflective in median survival estimates alone.

No statistically significant differences in survival were observed across primary tumour sites (oesophagus, GOJ, gastric) or histological subtypes in our cohort, although a trend toward longer OS was observed among patients with SCC (18 months) compared with AC (13 months). This observation is consistent with findings from KEYNOTE-590 [13], in which the survival benefit of pembrolizumab plus chemotherapy was particularly pronounced among patients with oesophageal SCC and higher PD-L1 expression. More specifically, as per strict NICE guidelines, there was no difference between outcomes of patients with oesophageal cancer (14 months) and GOJ or gastric cancer (16 months) with palliative chemo-IO (*p* = 0.734). These findings support the use of chemo-IO across anatomically diverse upper GI malignancies while suggesting potential biological differences in responsiveness between histological subtypes.

Following the start of chemo-IO, patients achieving radiological disease control (complete response, partial response, or stable disease) demonstrated substantially longer survival compared with those with interval disease progression. Median OS was 17 months (95% CI: 14.0–20.0) among patients with disease control compared with 4 months (95% CI: 2.1–5.9) among patients with progressive disease (*p* < 0.001). However, this observation should be interpreted cautiously, as radiological response represents an on-treatment outcome rather than a baseline prognostic factor and may therefore be subject to guarantee-time bias. Patients must survive long enough to undergo imaging and response assessment, meaning that those with early clinical deterioration or rapid disease progression are inherently less likely to be classified as having disease control. Consequently, the association observed here should be interpreted as descriptive rather than causal. The marked survival difference therefore likely reflects underlying disease biology and treatment response rather than an independent prognostic effect. Similar associations between objective response and long-term outcomes have been reported in pivotal trials including CheckMate-649 [16] observed with chemo-IO combinations. In univariate analysis, a greater number of IO cycles was associated with improved overall survival. However, this finding is likely influenced by immortal time bias and should not be interpreted as a causal relationship, patients who respond effectively receive further cycles of treatment. As such, treatment exposure is dependent on survival time, and the observed association reflects this time-dependent relationship rather than a direct therapeutic effect of additional cycles. Accordingly, duration-based metrics in this analysis should be interpreted as reflections of treatment tolerance and disease biology rather than independent prognostic factors. Similar limitations have been described in retrospective analyses of immunotherapy across multiple tumour types. While exploratory, this observation highlights the clinical importance of optimising management of irAEs and maintaining treatment continuity in patients deriving clinical benefit where feasible.

Immune-related adverse events occurred in 40.8% of patients in this cohort, with the majority being grade 1–2 in severity. Grade ≥ 3 toxicities were observed in 13.2% of patients, and two treatment-related deaths occurred (2.6%). The most frequent immune-mediated toxicities included hepatitis, colitis, and endocrine dysfunction. Overall, the spectrum of immune-related toxicities observed was consistent with that reported in clinical trials of chemoimmunotherapy in oesophago-gastric cancers. In the phase III KEYNOTE-590 [13] and CheckMate-649 trials [16], grade ≥ 3 treatment-related adverse events occurred in approximately 59–72% of patients receiving chemoimmunotherapy, although these figures include both chemotherapy-related and immune-mediated toxicities. When considering immune-specific toxicities alone, reported rates are substantially lower and broadly comparable with those observed in our cohort. Differences between trial and real-world toxicity rates may reflect variations in reporting practices, retrospective data capture, and differences in patient monitoring within routine clinical care.

Emerging evidence in gastrointestinal cancers suggests that the development of irAEs may correlate with improved treatment outcomes. A recent meta-analysis [25] including 22 retrospective studies of patients with gastrointestinal malignancies treated with immune checkpoint inhibitors (*n* = 2935) demonstrated that the occurrence of irAEs was associated with significantly improved OS (HR 0.45, 95% CI 0.36–0.57) and progression-free survival (HR 0.44, 95% CI 0.34–0.57). These findings are consistent with the hypothesis that immune-mediated toxicities may serve as a surrogate for enhanced immune activation and anti-tumour response. Nevertheless, the relationship between irAEs and survival is complex. Apparent survival advantages may be subject to time-dependent bias, as patients who remain on therapy longer have greater opportunity to develop irAEs. Evidence from multiple cancer types suggests that this potential survival benefit is largely driven by low-grade irAEs, whereas severe toxicities necessitating treatment interruption or discontinuation may not confer the same prognostic advantage [26]. In our cohort, neither the presence nor the severity of irAEs was significantly associated with OS or PFS. However, the small number of high-grade and fatal events limits the precision with which the impact of severe immune-related toxicity on survival can be determined.

Exploratory analyses suggested possible associations between baseline inflammatory markers and survival outcomes, including lymphocyte count and neutrophil–lymphocyte ratio (NLR). However, given the modest sample size and limited statistical power, these findings should be interpreted cautiously. NLR has been investigated as a prognostic biomarker in patients receiving immune checkpoint inhibitors across multiple tumour types, including oesophago-gastric cancers [27,28,29,30,31]. However, heterogeneity in proposed cut-offs and the absence of prospective validation currently limit its clinical utility [32,33,34,35,36].

This analysis has several strengths, including the use of multi-centre, real-world data reflecting routine clinical practice and the inclusion of both AC and SCC histologies. The availability of detailed staging information and toxicity data also provides important clinical context for interpreting outcomes. The multi-centre design and inclusion of patients treated according to NICE eligibility criteria enhance the generalisability of these findings to routine UK clinical practice.

However, several limitations should be acknowledged. Selection bias is an inherent limitation of this retrospective analysis. Patients included in this analysis were those considered suitable to receive combination chemoimmunotherapy in routine practice, which may exclude individuals with poorer performance status or significant comorbidities who are underrepresented in clinical datasets. In addition, data collection relies on the completeness and accuracy of electronic medical records. Although key clinical variables were available for most patients, missing data for certain laboratory parameters and subsequent treatment lines limited the scope of some analyses and may influence interpretation of survival outcomes.

Due to the absence of prospectively data collection, subjective irAEs (e.g., fatigue) may have been under-reported at low grades. All adverse events that led to further investigations, treatment, or modification of anti-cancer therapy were included. Data regarding subsequent lines of therapy were incomplete, which may influence interpretation of OS outcomes. A small cohort of mismatch repair (MMR)-deficient patients was identified, limiting the assessment of MMR as a prognostic indicator.

Radiological outcomes were based on consultant radiologist reports generated during routine clinical care rather than formal RECIST-based assessments. While this reflects real-world practice, variability in imaging interpretation and timing represents an inherent limitation of retrospective analyses. However, variability in radiological interpretation and imaging intervals represents an inherent limitation of retrospective real-world analyses.

Given the limited sample size and number of outcome events, multivariable Cox regression modelling was not performed. As a result, the reported associations from univariate analyses may be influenced by unmeasured confounding factors and should therefore be interpreted as exploratory. Finally, the modest sample size limits statistical power for subgroup analyses and increases the risk of type II error. As a result, several observed trends, particularly in exploratory biomarker analyses, should be interpreted as hypothesis-generating rather than definitive.

Clinically, these findings reinforce that chemo-IO represents an effective first-line treatment strategy for advanced upper gastrointestinal cancers, with survival outcomes broadly comparable to those reported in randomised trials including KEYNOTE-590 [13], CheckMate-649 [16], and KEYNOTE-859 [15]. Continued vigilance for immune-related adverse events remains essential given the potential for rare but serious toxicities, and prompt recognition with guideline-directed management is critical. In particular, effective management of less severe toxicities allowing patients to access further IO cycles. The strong association between radiologic response and survival further highlights the importance of early restaging to guide treatment continuation or modification.

Future prospective studies and collaborative real-world registries should aim to validate these observations, incorporating comprehensive biomarker profiling to better identify patients most likely to benefit from immunotherapy-based combinations. Additionally, they should clarify the relationship between irAEs, management strategies—including corticosteroid and immunosuppressant use—and long-term clinical outcomes.

## 5. Conclusions

To our knowledge, this represents the first UK multi-centre real-world study evaluating survival outcomes, immune-related adverse events, and exploratory prognostic biomarkers in patients receiving first-line palliative chemoimmunotherapy for oesophago-gastric cancers. Chemoimmunotherapy demonstrated survival outcomes comparable to those reported in pivotal randomised trials, with manageable toxicity profiles. Radiological disease control was associated with improved survival compared with early disease progression. However, the relatively small cohort size limits the ability to reliably identify prognostic and predictive biomarkers, particularly in relation to immunotherapy-related toxicity. Additionally, the retrospective design introduces potential selection bias and population heterogeneity, which should be considered when interpreting these findings.

Further multi-centre collaborative studies, ideally prospective and involving larger patient cohorts, are warranted to validate these findings and enable robust subgroup analyses. Such studies would also facilitate the identification and validation of prognostic biomarkers, allow more precise assessment of the efficacy of palliative chemoimmunotherapy, and improve prediction of immune-related adverse events in upper gastrointestinal malignancies.

## Figures and Tables

**Table 1:** The overall survival (OS) and progression-free survival (PFS) results of the key randomised controlled trials investigating chemoimmunotherapy (chemo-IO) in OG cancers showing superiority compared with chemotherapy alone with the current NICE approved PD-L1 combined positive score/tumour proportion score thresholds.

Trial	Cancer	Immunotherapy	Total Patients	Chemo-IO OS/PFS (Months)	Chemo OS/PFS (Months)
KEYNOTE-590 [13]	Oesophageal	Pembrolizumab	749	13.9/7.5	8.8/5.5
CheckMate-648 [14]	Oesophageal SCC	Nivolumab	970	15.4/6.9	9.1/4.4
KEYNOTE-859 [15]	Gastric	Pembrolizumab	1579	13/6.9	11.4/5.6
CheckMate-649 [16]	OG AC	Nivolumab	1581	14.4/7.7	11.1/6.1

SCC, squamous cell carcinoma; OG, oesophago-gastric; AC, adenocarcinoma.

**Table 3:** Immunotherapy-related adverse event profile and severity of the patients treated with chemoimmunotherapy. In total, 31 patients experienced a total of 34 toxicities; 3 patients had multiple toxicities recorded.

Toxicity	N	%
**Neuropathy Grade**		
1	1	1.3%
2	3	3.9%
**Nephritis Grade**		
2	2	2.6%
**Hepatitis Grade**		
1	3	3.9%
3	2	2.6%
5	1	1.3%
**Pneumonitis Grade**		
2	1	1.3%
3	1	1.3%
**Colitis Grade**		
1	1	1.3%
2	4	5.3%
3	1	1.3%
4	1	1.3%
**Dermatitis Grade**		
2	1	1.3%
3	3	3.9%
**Myositis Grade**		
2	1	1.3%
**Meningoencephalitis Grade**		
5	1	1.3%
**Myalgia Grade**		
2	1	1.3%
**Hypothyroidism Grade**		
1	5	6.6%
**Endocrinopathy Grade**		
2	1	1.3%

## Data Availability

The data presented in this study are available on request from the corresponding author due to NHS data protection policies.
